# An Organised Boycott

**Published:** 1898-10-01

**Authors:** 


### AN ORGANISED BOYCOTT.

At a recent meeting of the South Australian Branch of the British Medical Association, notice was given of a motion the terms of which should receive the careful consideration of members of other branches in other parts of the world. It runs as follows: "That the Council be respectfully requested to take such steps as may be necessary for securing the removal from the membership of the Branch of those members who continue in association with men who have been expelled from the British Medical Association."

Clearly the intention of this resolution is to give additional force to the penalty of expulsion which the British Medical Association some time ago inflicted upon some of its members. The important point is, however, that it seeks to set up the doctrine that a man expelled from the British Medical Association is to be looked upon as so tainted in a professional sense, so utterly beyond the pale, that mere association with him is to be regarded as giving good ground for deprivation of membership.

This, as it seems to us, is going a long way, and we feel sure that if such a resolution is carried, and is acted upon with all the petty cliquishness that is apt to prevail in provincial centres, its effect must be to create a widespread feeling of unrest among the members of the Association. The expulsion of a couple of men at the Antipodes, no doubt, seemed a small affair to many who partook in that very dubious transaction, but when we find it proposed that association with an expelled member is to be taken as ground for expulsion from a branch, we may fairly ask whether association with persons so expelled is also in turn to be taken as ground for another series of expulsions, and whether this is to go on like a "snowball" begging letter? We do not wish to go back to the original offence which led to the expulsion of two members of the Association resident in South Australia, but the bringing forward of the resolution we have referred to, which shows how far-reaching in its effects such a sentence of expulsion may become, renders it necessary that we should direct attention to the nature of the tribunal to which every medical practitioner when he accepts the membership of the British Medical Association submits his professional honour, and to the character of the proceedings at the final court of appeal, by which his professional future may have to be decided if he should happen so to offend the practitioners in his neighbourhood as to have a resolution for his expulsion levelled against him.

Anyone who has attended the general meetings of the British Medical Association, and has taken note of the noise and excitement by which they are too often characterised, and of the cries of "Vote! vote! vote!" when any quiet member tries to put forward views that are not generally acceptable, must be well aware that such a meeting is the very last body, the worst fitted that could be imagined, to decide a question of such serious moment as the expulsion of a member. It may be said, however, that such a question would never come before a general meeting until the member had been already expelled by the Council, and that so great is the trust and confidence which is universally felt in the Council, that no one need hesitate to place himself, his honour, and his prosperity entirely in their hands.

But public bodies acting collectively are curiously apt to be swayed by other than judicial considerations, and it is impossible to forget that according to the officially published account, the reason for which the two members we have referred to were expelled was not that they had done anything morally, ethically, or professionally wrong, but that they had taken office at the Adelaide Hospital "at a time when the whole staff of that hospital had resigned in consequence of the conduct of the Government towards the governing body and the staff, and when no efficient persons were to be found in South Australia to take their places, such was the general sympathy and support which that staff met with." And for no other reason. Special emphasis was laid upon the fact that this was the sole reason, so that we must believe that the Council did not go into the question whether the South Australian branch was right or wrong, but was content to pass a resolution of expulsion "solely" because these men had acted in opposition to the expressed wishes of the practitioners of the place.

Let us for once call things by their right names. These two men were expelled because they were "blacklegs." We do not say this was wrong. The whole thing was no doubt perfectly legal. Any association can choose its associates. But this was clearly the setting up of a new criterion of professional rectitude, and we do not think that a Council which could allow itself to take such a course can be relied upon to afford a man any protection against popular clamour.

When, then, one remembers that the only court of appeal from such a tribunal is an excited meeting, sitting with closed doors and without reporters, and that an attempt is now being made to organise a professional boycott against all who so offend their brethren as to get expelled, we think many will ask themselves whether membership is worth the risk, especially as the journal which is the backbone of the Association can be got by anyone who choses to pay for it.

